# Building Pathogen Genomic Sequencing Capacity in Africa: Centre for Epidemic Response and Innovation Fellowship

**DOI:** 10.3390/tropicalmed10040090

**Published:** 2025-03-31

**Authors:** Eric Agboli, Molalegne Bitew, Christian N. Malaka, Tiangay M. P. S. Kallon, Alhaji M. S. Jalloh, Baron Yankonde, Doreen M. Shempela, Jay F. M. Sikalima, Mutale Joseph, Mpanga Kasonde, Feleke M. Demeke, Ayemfouo F. I. Valdese, Lele B. Grace, Godwe Célestin, Ana Papkiauri, Sado Y. F. Berlange, Janet Majanja, Vane K. Omwenga, Evalyne N. Wambugu, Samuel M. Kariuki, Alex A. Mwanyongo, Ubheeram Jaykissen, Caroline Abanto Alvarez, Seyni Ndiaye, Benjamin Moswane, Ester K. Adamson, Mariam Makange, Luka Sote, Ibrahimm Mugerwa, Julius Sseruyange, Patrick Semanda, Brian A. Kagurusi, Abdualmoniem O. Musa, Kourush Fassihi, Lavanya Singh, Monika Moir

**Affiliations:** 1 Department of Biomedical Sciences, School of Basic and Biomedical Sciences, University of Health and Allied Sciences, Ho PMB 31, Ghana; 2 WHO Collaborating Centre for Arbovirus and Haemorrhagic Fever Reference and Research, Bernhard Nocht Institute for Tropical Medicine, 20359 Hamburg, Germany; 3 Health Biotechnology Directorate, Bio and Emerging Technology Institute of Ethiopia, Addis Ababa P.O. Box 5954, Ethiopia; molalegne23@yahoo.com; 4 Institut Pasteur of Bangui, Bangui 923, Central African Republic; christian.malaka@pasteur-bangui.cf; 5 Viral Hemorrhagic Fever Laboratory, Kenema Government Hospital, Kenema 232, Sierra Leone; tkallon03@gmail.com (T.M.P.S.K.); alhajiboumaloljalloh@gmail.com (A.M.S.J.); 6 Churches Health Association of Zambia, Lusaka P.O. Box 34511, Zambia; baron.yankonde@chaz.org.zm (B.Y.); doreen.shempela@chaz.org.zm (D.M.S.); jay.sikalima@chaz.org.zm (J.F.M.S.); 7 Zambia National Public Health Institute, Lusaka P.O. Box 10101, Zambia; mutalejoseph48@gmail.com (M.J.); mpanga.kasonde@gmail.com (M.K.); 8 Department of Medical Laboratory Sciences, College of Medicine and Health Sciences, Bahir Dar University, Bahir Dar P.O. Box 79, Ethiopia; bernabas2007@gmail.com; 9 Department of Biochemistry, Faculty of Science, University of Yaoundé I, Yaoundé P.O. Box 812, Cameroon; ayemfouoironne@gmail.com (A.F.I.V.); lelebrendag@gmail.com (L.B.G.); 10 Center for Research on Emerging and Re-Emerging Diseases, Yaoundé P.O. Box 906, Cameroon; godwec@gmail.com; 11 National Center for Disease Control and Public Health, Tbilisi 0198, Georgia; a.papkiauri@ncdc.ge; 12 Centre for Research in Infectious Diseases, Department of Microbiology and Parasitology, Yaoundé P.O. Box 13591, Cameroon; francine.sado@crid-cam.net; 13 Kenya Medical Research Institute, Nairobi P.O. Box 54840-00200, Kenya; jmajanja@gmail.com (J.M.); vaneomwenga9@gmail.com (V.K.O.); 14 Department of Biological Sciences, School of Pure and Applied Sciences, University of Embu, Embu P.O. Box 6-60100, Kenya; wambuguenyambura@gmail.com; 15 Department of Biological Sciences, University of Eldoret, Eldoret P.O. Box 1125-30100, Kenya; skariuki@uoeld.ac.ke; 16 Public Health Institute of Malawi, Lilongwe 3, P.O. Box 30377, Malawi; alexmwanyongo@gmail.com; 17 Ministry of Health and Wellness, Port-Louis 11328, Mauritius; ubheejay@gmail.com; 18 Facultad de Ciencias e Ingeniería, Universidad Peruana Cayetano Heredia, Lima 150135, Peru; caroline.abanto.a@upch.pe; 19 Institut de Recherche en Santé de Surveillance Épidémiologique et de Formation, Pole Urbain de Diamniadio, Dakar BP 7325, Senegal; seynindiaye07@gmail.com; 20 Department of Medical Microbiology, School of Pathology, University of the Free State, Bloemfontein 9301, South Africa; benjaminmoswane@gmail.com; 21 SACIDS Foundation for One Health, Morogoro P.O. Box 3297, Tanzania; ester.adamson@sacids.org (E.K.A.); mariam.makange@sacids.org (M.M.); 22 Mbeya Zonal Referral Hospital Reference Laboratory, Mbeya P.O. Box 259, Tanzania; soteluka5@gmail.com; 23 Ministry of Health of Uganda, Kampala P.O. Box 7272, Uganda; mugerwaibrahim@gmail.com; 24 National Health Laboratory and Diagnostic Services, Kampala P.O. Box 7272, Uganda; juliuseruyange@gmail.com (J.S.); semandapatrick@gmail.com (P.S.); abkagurusi@gmail.com (B.A.K.); 25 Department of Microbiology, Faculty of Medical Laboratory Sciences, University of Kassala, Kassala 31111, Sudan; mo.omer1991@gmail.com; 26 Kwazulu-Natal Research Innovation and Sequencing Platform, University of KwaZulu-Natal, Durban 4001, South Africa; kourushfassihi@gmail.com (K.F.); singhl@ukzn.ac.za (L.S.); 27 Centre for Epidemic Response and Innovation, School for Data Science and Computational Thinking, Stellenbosch University, Stellenbosch 7600, South Africa

**Keywords:** bioinformatics, capacity building, genomic surveillance, lessons, pathogens, training

## Abstract

The World Health Organization African region has the greatest infectious disease burden in the world. However, many African countries have limited capacity to rapidly detect, report, and respond to public health events. The Centre for Epidemic Response and Innovation (CERI), KwaZulu-Natal Research Innovation and Sequencing Platform (KRISP) in South Africa, and global Climate Amplified Diseases and Epidemics (CLIMADE) consortium are investing in building the capacity of African scientists in pathogen genomics and bioinformatics. A two-week long (11–21 April 2023) intensive training in wet-laboratory genomic data production, bioinformatics, and phylogenetic analyses of viral and bacterial pathogens was held in Cape Town, South Africa. Training was provided to 36 fellows with diverse backgrounds from 16 countries, 14 of which were low- and middle-income African countries. In this report, we, the fellows, share our collective experiences and describe how the learnt skills have been integrated into the operations of our home institutions to advance genomic surveillance capabilities. We identified the in-person and hands-on learning format of the training, taught by genomics experts and field application specialists, as the most impactful elements of this training event. Adaptation and miniaturisation of protocols to detect other pathogens is a great enhancement over the traditional method of using a single protocol for a pathogen. We note the duration of the training as the largest limiting factor, particularly for the computationally intensive bioinformatics sessions. We recommend this programme continue to build pathogen genomics capacity in Africa.

## 1. Introduction

April 2023 marked the 20th anniversary of the completion of the sequencing of the first human genome, coinciding with the genomics and bioinformatics training on pathogen sequencing for African fellows held in Cape Town, South Africa. Since the initiation of the human genome project, we have witnessed major advances in genomic sequencing technologies [1] to the extent that whole-genome sequencing of pathogens is now routine for disease genomic surveillance [2,3,4]. Pathogen genomic sequencing has become pivotal in disease surveillance, early detection and diagnosis, drug design, and clinical care [1]. Coupled with the impressive progress in generating genomic data is the growing need for bioinformatics skills to process and analyse these large biological datasets. However, in resource-limited settings such as low- and middle-income countries in Africa, major challenges continue to persist, such as a lack of access to local bioinformatics expertise and training, lack of computational infrastructure, and internet access instability [5].

During the recent COVID-19 pandemic, low- and middle-income countries were initially sequencing SARS-CoV-2 genomes from a substantially smaller proportion of COVID-19 cases, with a slower turnaround time, than high-income countries [6]. This was due to a myriad of reasons, some of which include the lack of coherent national policies, a limited number of well-trained genomic scientists, poor research infrastructure, and socioeconomic challenges [7]. However, subsequent major investments in SARS-CoV-2 sequencing capacity from the Africa Centres for Disease Control and Prevention (Africa CDC) and the World Health Organization Regional Office for Africa (WHO AFRO) exponentially increased the number of sequences generated by Africa [8]. Increased genomic surveillance efforts were pivotal in the pandemic response, as this allowed public health officials in African countries to track the pathogen, understand its evolution, and better navigate local and national outbreaks.

Following the successful drive to upscale African pathogen genomic sequencing capacity [9], there remains a need to continually support these activities via skills transfer to continue to track the SARS-CoV-2 evolution and respond to emerging epidemics in Africa. Two partnering institutions, Kwazulu-Natal Research Innovation and Sequencing Platform (KRISP) at the University of KwaZulu-Natal and the Centre for Epidemic Response and Innovation (CERI) at the University of Stellenbosch, both of which are specialised genomic sequencing centres of the Africa Pathogen Genomics Initiative (Africa PGI) and supported by the Africa CDC and WHO AFRO, remain committed to genomics capacity-building in Africa. The vision of CERI and KRISP is to effectively respond to epidemics through pathogen genomics surveillance; enhance biomedical discovery; improve the treatment, diagnosis, and prevention of human disease; and generate socioeconomic opportunities for Africa. Spawned from CERI is the Climate Amplified Diseases and Epidemics (CLIMADE) global consortium. The CLIMADE consortium recognises the immediate threat of climate-sensitive diseases and works to generate knowledge and develop tools to predict, track, and control these diseases in the most affected countries in the world. CLIMADE commends the historical experiences of the Global South in responding to outbreaks and epidemics of infectious diseases [10,11]. CLIMADE initiatives encourage the Global South to take the lead on the global public health response for future epidemics and pandemics. The CLIMADE Africa Working Group is one such initiative: a continental consortium working to fill critical knowledge gaps of climate-amplified disease transmission predicted to pose threats to the health of people in Africa.

Beyond the scientific objectives, CERI, KRISP, and CLIMADE work to develop young African scientists to stimulate research capacity and sustainability in Africa. Succeeding nine successful training workshops hosted by CERI and KRISP, together with CLIMADE, an educational initiative of genomics and bioinformatics training covering wet- and dry-laboratory components of pathogen sequencing was created. Here, we, the fellows, have collectively written this report to share our experiences from the workshop, chart the benefits of the training, and describe the challenges encountered. One fellow, Dr. Molalegne Bitew, reports how he has integrated the transferred skills from the training to the operations of his home institution, and, together, we pose a way forward for fellows to further genomics and bioinformatics in Africa.

## 2. Programme Description

### 2.1. Selection of Fellows

The training opportunity was advertised by CERI on several platforms: the CERI website and social media, and networks of the CLIMADE Africa Working Group, Abbott Pandemic Defence Coalition, and United World Antiviral Research Network (UWARN). Applications were competitive, and as such, we had to detail our qualifications and levels of expertise for laboratory procedures and bioinformatics workflows, describe relevant past training opportunities already attended, and provide a motivation for the award of a fellowship. We rated our skill levels in wet-laboratory protocols and bioinformatics abilities using the information provided in the application. A total of 117 applications were received by CERI, which were scored by three in-house reviewers, from which 32 of the highest-rated applications were selected. A further four fellows participated in the training with independent funding from the WHO.

**Figure 1:**
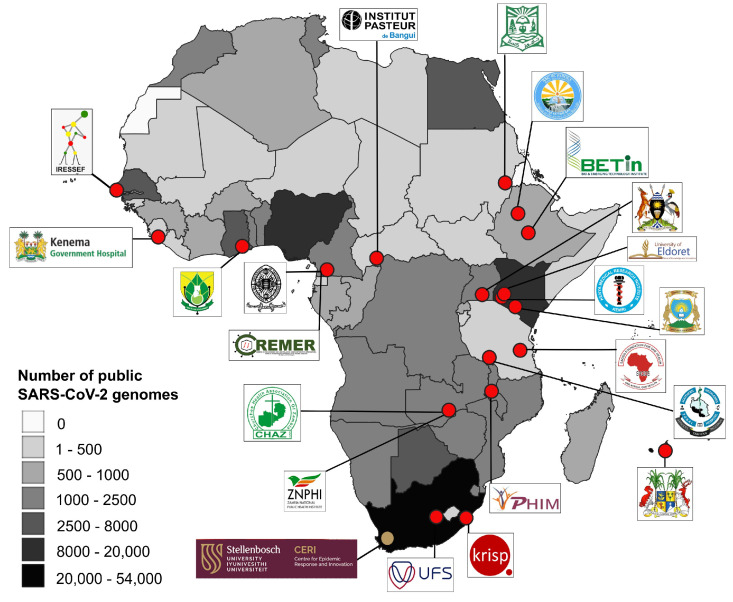
Map of Africa depicting the location and affiliation of the fellows who participated in the training (red circles). The location of CERI, where the training was hosted, is shown with a gold circle. Country colour displays the number of SARS-CoV-2 genomes publicly available on the Global Initiative on Sharing All Influenza Data (GISAID) as a proxy for pathogen genomic sequencing capacity (data accessed on 17 July 2023). Map produced with Quantum GIS [12].

The fellows represented 14 African countries (Figure 1), Georgia, and Peru. We represented countries with a range of pre-existing pathogen genomic sequencing capacities. The number of SARS-CoV-2 genomes, publicly available from GISAID, was used in Figure 1 as a proxy to depict the general sequencing capacity of each African country. Nine fellows represented countries with fewer than 1000 public SARS-CoV-2 genomes, and sixteen were from countries that had produced fewer than 8000 genomes.

The training was generously funded by the Abbott Pandemic Defence (APDC) Coalition, WHO AFRO, African Society for Laboratory Medicine (ASLM), Africa CDC, and the Rockefeller Foundation. All expenses associated with travel, accommodation, meals, and attendance were covered by the funders.

### 2.2. Learning Objectives of the Training

The training was held from 11 to 21 April 2023, and the programme was designed to cover the process of generating and analysing genomic data, from amplification of pathogen nucleic acids to communicating scientific results to non-specialists for public health response. The programme was designed such that fellows may practically implement disease genomic surveillance methods upon return to home institutions. As such, the learning objectives were to develop skills in the following:

1. Hands-on training in wet-bench protocols, including nucleic acid amplification, library preparation, and sequencing.
2. Adaptation of protocols to sequencing other viral and bacterial pathogens.
3. Pathogen genomic bioinformatics, which included an introduction to foundational computational skills, base-calling and sequence assembly, sequence quality control checks, and submission of sequence data to public genomic repositories.
4. Basic phylogenetic analysis, which included compiling a sequence alignment and building phylogenetic trees.
5. Reporting sequencing results and communicating scientific findings for public health applications.

### 2.3. Fellows’ Professional Profiles

Our range of molecular biology expertise was varied, as we have different occupations: Laboratory Technologists, Post-graduate students, Post-doctoral Research Fellows, Bioinformaticians, Clinical Infectious Diseases Specialists, Public Health Officials, and Senior Scientists, with 48% employed at public health institutions and 52% employed by universities or non-profit research centres. All the fellows hold Bachelor’s degrees, predominantly in the fields of Biological and Biomedical Sciences, with 41% trained to the Master’s degree level and 15% holding PhD degrees. Prior to attending this training, 14% of us had no training in genomics or bioinformatics. While 43% had undergone formal training in genomics and/or bioinformatics, 23% had self-studied, and 20% had both self-study and formal training in genomics and/or bioinformatics.

**Figure 2:**
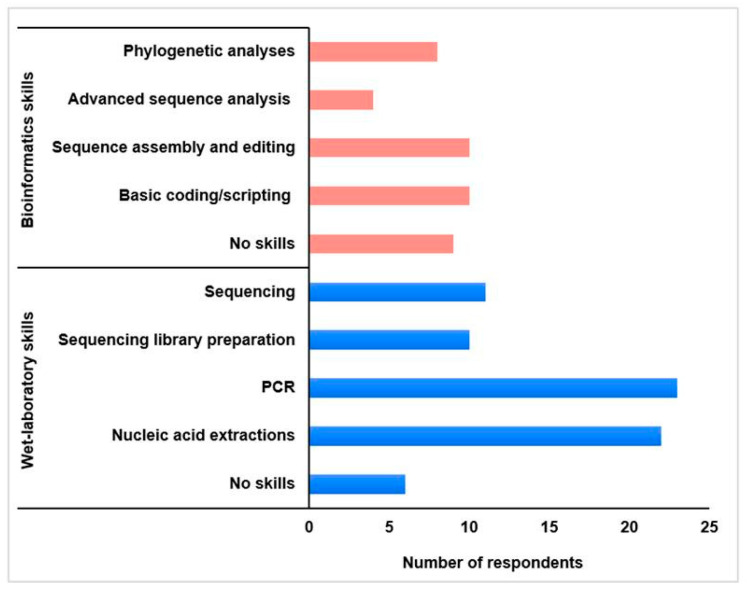
Rating of fellows’ bioinformatics (red) and wet-laboratory (blue) skills. The number of respondents depicts the number of fellows possessing each skill prior to attending the training opportunity (PCR: polymerase chain reaction).

Before attending the training, our skill levels in wet-laboratory protocols and bioinformatics abilities (Figure 2) were rated. CERI used this in the selection process not only to identify fellows who would benefit from the training, but also to include individuals with some pre-existing skills so that fellows may learn from one another. We mostly reported having expertise in the wet laboratory, particularly in nucleic acid extractions and polymerase chain reaction methods (~80%). Approximately 60% required training on library preparation and sequencing methods, while our greatest need for upskilling was in bioinformatics and phylogenetic analyses.

### 2.4. The Training Event

The training covered viral and bacterial adaptive sequencing and bioinformatics protocols. During the training, we spent approximately 42.5 h learning wet-laboratory protocols, 5 h on basic computational skills (introduction to Unix, bash scripting, R and RStudio), and 19 h learning bioinformatics expertise (base-calling and assembly, sequence assembly and analysis, sequence quality control, sequence submission to public databases, and basic phylogenetics). Hence, a total of 24 h was used for bioinformatics training (Table 1). We were trained to sequence whole genomes of SARS-CoV-2 on the Illumina COVIDSeq protocol and how to adapt this protocol to other viral pathogens. Additionally, we were trained on whole-genome sequencing of *Mycobacterium tuberculosis* bacteria (MTB) with the miniaturised version of the Illumina DNA Prep protocol [13], and how to use this protocol for sequencing of *Vibrio cholerae* bacteria. For the bioinformatics components, we were taught how to base-call and assemble genomes, edit genomes and apply quality control steps, identify viral lineages, and compile phylogenetic trees. We also gained skills on how to graph sequencing results and phylogenetic trees to report the main findings for public health applications.

The training coincided with the inauguration of Stellenbosch University’s Biomedical Research Institute (BMRI). This facility is the most advanced biomedical research complex on the African continent. This facility is used for research on the genetic and biomolecular basis of diseases afflicting South Africa and the rest of the African continent [14]. We attended several events, laboratory tours, and scientific seminars associated with the inauguration. One event of particular interest to us was the full-day Genomics Symposium, wherein talks were given by local and international genomics researchers and industry leaders.

### 2.5. Approaches to Gather Fellows’ Perspectives

The first two authors conceived this manuscript after completing the training. The design and writing were led by these authors, which began within a few weeks of the conclusion of the training. We obtained fellows’ perspectives on the training via an online questionnaire (semi-structured interviews) in December 2023 (Supplementary Materials S1 and S2), to which 32 fellows responded. We collated and summarised the anonymised answers to quantitatively and qualitatively capture the fellows’ perceived outcomes of the training. Data were analysed using Microsoft Excel.

## 3. Discussion

This section details the fellows’ perspectives on the training outcomes, critical evaluation of the training programme, knowledge transfer at home institutes, and the way forward for Africa.

### 3.1. Fellows’ Perspectives on the Training Outcomes

#### 3.1.1. Acquisition of Specialised Skills and Knowledge Advancement

Generally, we perceived this training opportunity to have offered the required specialised expertise in genomic sequencing and bioinformatics analyses of the viral and bacterial pathogens studied. When asked, the other 32 fellows agreed that attending the training transformed the way their institutions monitor disease outbreaks. Twenty-one fellows stated that learning the approach of disease genomic surveillance, and the use of the specific methods and tools to execute this work, upskilled them to be capable of monitoring disease outbreaks with genomic surveillance strategies. One fellow described how “the integration of wet-lab and dry-lab techniques has provided a well-rounded perspective, making our surveillance efforts more efficient, accurate, and adaptable to the evolving landscape of infectious diseases”.

When asked if fellows felt the learning objectives of the training were met, 20 responded in the positive. Two fellows indicated that they thought the objectives were partially met due to insufficient time allocated for all of the topics covered, with ten fellows echoing this statement, along with the need for more time and hands-on practice for bioinformatics tools and methods. An insightful comment from one fellow stated, “Yes, the objectives were met as the session covered DNA library preparation, sequence quality checking and trimming using Trimmomatic, sequence alignment with AliView, and phylogenetic analysis using RStudio (Version 4.2.2). Command-line usage was introduced, but insufficient time limited learning, though resources were provided for further study”. Overall, we judged the training to have had a significant positive impact on our abilities to conduct genomic surveillance in response to outbreaks, a strategy rarely used in some regions of the continent. However, several months following the conclusion of the training, a fellow raised the issue that implementation of the learnt material was not yet effective at their institution. The limitations to implementation require further investigation.

#### 3.1.2. Revolution of Next-Generation Sequencing (NGS)

We learnt how NGS has revolutionised disease surveillance and outbreak investigations. Indeed, NGS does not only permit clinical diagnosis but has also proved useful in other medical applications, such as determining disease risk, therapeutic identification, prenatal testing, and cancer and autoimmune disease testing [15]. One fellow noted that learning how to perform whole-genome sequencing was useful within their institution to inform patient treatment strategies for multidrug-resistant tuberculosis infections. The theory and practical skills learnt during this training empower us to expand our research portfolios to other fields in which NGS techniques are employed.

#### 3.1.3. Genomic Surveillance of Pathogens: Real-Time Detection and Results Communication

Genomic data are useful for monitoring the localised, regional, and global transmission of individual pathogen strains, which is paramount for disease intervention strategies. Disease and genomic surveillance systems are generally regarded as weak in low- and middle-income countries in Africa, with most African countries implementing integrated disease surveillance and response strategies to varying degrees [16]. However, we embrace using genomic technologies in health surveillance settings. We recognise the substantial upscaling of genomics capacity that occurred during the SARS-CoV-2 pandemic, and we recognise that now is the time to leverage the systems and infrastructures that were developed to continue to expand and improve our abilities to detect and respond to infectious disease threats. We must maintain the momentum of developing genomics capacity in our countries and our continent. We view this training opportunity as timely to continue to stimulate genomic surveillance activities and recognise the need to shift our focus and protocols to other pathogens of public health concern. The experience gained from this training has encouraged us to look beyond disease diagnosis and provided us with insights to establish surveillance networks at our home institutions.

One of the last, and arguably one of the most important, steps of the pathogen genomic surveillance process is communicating relevant results to politicians, public health officials, and the public [2]. During the pandemic, this was achieved via specialist presentations, dashboards, and databases that relayed results in easy-to-understand graphics [17,18,19]. This training event had a strong emphasis on learning how to interpret the results of genomic data analyses, how to create visualisations of the data, and how to translate genomic sequencing results into practical and digestible information for non-specialists. During this training, we polished our science communication skills for our work to have practical applications in public health policies. Implementation of these newly learnt skills at our local institutions will surely have tangible impacts on our capacity to respond to disease outbreaks.

#### 3.1.4. Protocol Adaptation and Miniaturisation

The Illumina COVIDSeq protocol was originally developed to sequence SARS-CoV-2 genomes but has since been adapted for other viral pathogens [20,21]. Adaptation of a protocol for simple and scalable amplification and sequencing of other pathogens allows the world to more readily pivot its genomic surveillance response to the next pathogen of concern when it arises. We value having learnt the process to adapt the protocol in this training and feel it will greatly increase the future genomic capacities and breadth of impact of our laboratories. The techniques for protocol adaptation that we learnt include nucleic acid enrichment or extraction-free, target primer design; multiplexing; sequencing depths/read lengths; customisation of panels; and variant analysis. Protocols for respiratory pathogen panels can be adapted for SARS-CoV-2, influenza, respiratory syncytial virus (RSV), and common bacteria pathogens [2,13,22]. The protocol adaptation strategy effectively combines flexibility with the robustness of the Illumina COVIDseq platform, making it highly adaptable for extensive pathogen screening initiatives compared to the traditional targeted pathogen screening platforms.

Also, the optimisation of protocols to use much reduced volumes of reagents and reduce the time required to prepare sequencing libraries [13], without compromising the results, is commendable. This is a particularly valuable tool for institutions operating within resource-constrained settings, as it makes sequencing pathogen genomes more efficient and cost-effective. Having received training on an optimised protocol will allow us to more easily increase the number of genomes sequenced in our home institutions with a reduced turnaround time to produce them. This will greatly aid our local institutions in emergency preparedness for future outbreaks of a broad scope of diseases.

Perhaps the most important lesson learnt in this regard is the reminder that scientists need to be innovative in their approaches to solving problems. Indeed, the scientists at CERI provide an innovative platform for health professionals in biomedical research. Lastly, it is interesting to note that the CLIMADE consortium is keen on open sharing of pathogen sequencing laboratory protocols. CLIMADE has uniquely shared sequencing protocols for all to access (https://github.com/CERI-KRISP/CLIMADE/tree/master/Protocols, accessed on 18 April 2023) and use. In the long term, with a collaborative effort from many contributing institutions, a unified library of protocols for pathogen detection will be created to benefit Africa and the world. The protocol sharing through the CLIMADE consortium and skills obtained during the training will be beneficial in implementing a range of research projects at our home institutions.

#### 3.1.5. In-Person Networking and Collaboration

Bringing emerging scientists from different African countries together has provided a springboard for us to launch collaborative projects and develop professional connections with one another. The interactive sessions with trainers, field experts, companies, and sponsors also provided opportunities for networking and collaboration. As previously described, we attended the Genomics Symposium and other events of the inauguration of Stellenbosch University’s Biomedical Research Institute. The consensus from the fellows was that attending these events enriched the training experience in the following ways: by providing opportunities to learn new developments in the field from experts, gain exposure to laboratory arrangements, network with field leaders, provide a broader context for our training, and showcase real-world applications and the importance of our work. One fellow described how “the combination of both training and external events enriched the learning experience, offering a more holistic perspective on the field of bioinformatics and genomics”. However, it is important to note that some training participants may not find attending such extra events to be the most valuable use of time, as one fellow noted it had no impact on the training experience.

On the back of the pandemic, our workdays are still punctuated with many online meetings and workshops. However, this training was held in person, with obvious benefits for learning laboratory workflows by physically handling instruments and performing hands-on tasks. There was a resounding echo of positive comments when we asked fellows their opinions of attending an in-person training event rather than a virtual one. Overall, we felt it to be a more immersive and engaging experience, enhancing the depth of understanding facilitated by close contact with the trainers. We also felt inspired by working in a well-equipped laboratory. We valued the opportunity to network and form close working relationships with one another. One fellow based outside of the African continent noted that “in-person knowledge sharing and collaboration—especially within the African scientific community—were extremely exciting. During that programme, I was just one individual from Eurasia, but it was great to get to know and meet the African scientific community”.

Undoubtedly, this is the beginning of a collaborative era for African genomic scientists to take charge of ourselves to meet the challenges of mitigating the impacts of infectious diseases of public health importance. Going forward, we plan to partner more with CERI and KRISP, with collaborations already underway via the CLIMADE Africa Working Group; student exchange and postdoctoral researcher programmes; and sharing of skills, samples, reagents, and protocols. Following the lead of Professor Tulio de Oliveira of CERI, KRISP, and CLIMADE, we are inspired to take a leading role in genomics research and pathogen surveillance in our home institutions, countries, regions, and globally. We believe this is possible by establishing robust collaborative networks across Africa to facilitate innovative solutions for healthcare problems and elevate healthcare research on the continent.

### 3.2. Critical Evaluation of the Training Programme

#### 3.2.1. Novel Approaches

**Figure 3:**
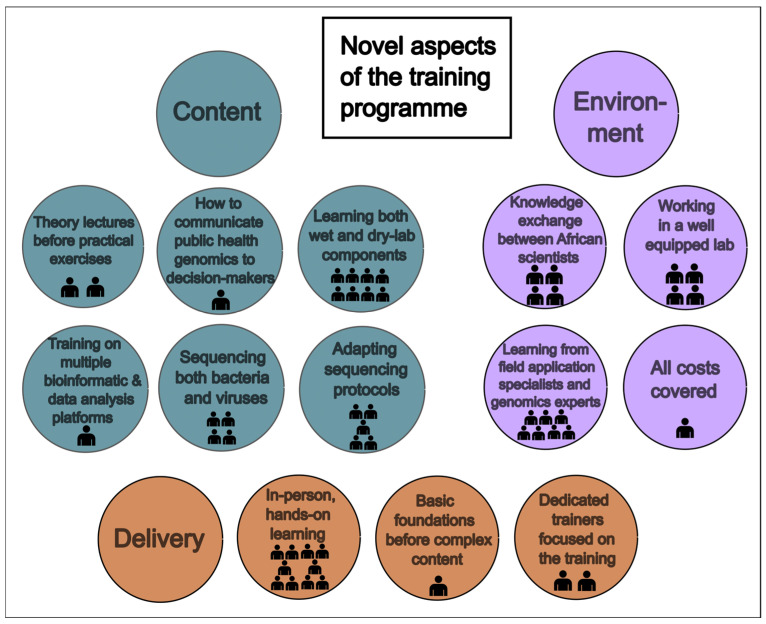
Novel aspects of the training are grouped into the categories of Content, Environment, and Delivery. The number of figure icons indicates how many fellows listed each aspect in their questionnaire responses.

Bearing in mind that, before this training, 14% of us had not attended formal training opportunities in genomics or bioinformatics, while 63% had some form of formal training (not including self-learning), we identified key aspects of this training event that were new or different from previous training we had attended (Figure 3). We believe other educational initiatives should try to replicate these aspects. We identified the in-person and hands-on learning opportunity, of both the wet- and dry-lab components of pathogen sequencing, from genomics experts and field application specialists as the most impactful approaches of this training event. One of the fellows provided this response to our anonymous questionnaire: “This training stood out from previous experiences in several ways, offering a balanced mix of wet-lab and dry-lab sessions, fostering a holistic understanding of genomics. This integration of hands-on experiments with bioinformatics analysis offered a comprehensive approach. The training environment promoted collaboration, allowing participants to apply theoretical knowledge in practical scenarios. This approach not only deepened understanding but also facilitated networking and knowledge exchange among participants. Overall, the unique combination of practical skills, interactive learning, and a collaborative environment made this training exceptionally effective. Other initiatives should consider replicating this blend to enhance engagement, practical application, and knowledge transfer”.

#### 3.2.2. Train-the-Trainer

This training event intended to broadly upskill genomics capacity across Africa, with a ‘Train-the-Trainer’ model, by providing the training in a way such that we are able to further disseminate the learning content as a trainer to colleagues in our home institutions and broader networks. When asked, 55% of the fellows felt that the training was sufficient to train others on the learning content, with one fellow having already conducted such training as the trainer. Approximately 27.5% were partially confident that they could host such training, with most being fully confident in wet-lab protocols but hesitant in their capabilities as a bioinformatics trainer. However, approximately 17% responded in the negative, noting the need for more practice with the methods and tools to be confident in providing such training, or needing further support from CERI and KRISP scientists to set up and conduct this training. One fellow commented “I would benefit from additional materials and perhaps a more structured follow-up plan to feel fully confident in training others. Some aspects could be refined to enhance my ability to effectively convey the content to a diverse audience. However, with continued support and resources, I believe I can successfully train others on the acquired content”. We regard this as a partially successful ‘Train-the-Trainer’ programme that may be improved by CERI and KRISP if a number of the limitations experienced during the training (described below) are addressed. It is then likely that future fellows will feel thoroughly equipped to further propagate the knowledge.

#### 3.2.3. Limitations and Suggestions for Improvement

Twenty-six fellows identified the duration of the training as the largest limiting factor. Due to our diverse backgrounds, with fellows mostly having experience in either the computational or wet-laboratory component, having only two weeks to bring all fellows to the same level on both components was challenging. This resulted in difficulties for some to follow a few sessions, particularly the computationally intensive bioinformatics sessions. We recommend ameliorating this challenge in the following ways: extend the training for bioinformatics to two weeks or the full training period up to four weeks, with more time for hands-on practice of methods; provide learning material on computational basics of bioinformatics to prepare in advance of the training; provide all learning materials (for both wet- and dry-lab) before the training; reduce the number of tools taught for the bioinformatics component to narrow the focus; provide homework exercises for repeated practice and learning after contact sessions; and lastly, present the bioinformatics analysis in the mornings and schedule the activities that require less concentration in the afternoons for when we are fatigued.

Tying into this limitation is the recommendation that the knowledge needs of the fellows be assessed beforehand and that fellows be grouped based on their prior capabilities. The training content and schedule may then be tailored to the specific needs of the different groups of fellows so that more time may be spent on certain components where skills are lacking. Also, we recommend the training programme be designed collaboratively between trainers and trainees to reach a consensus on the content presented. For example, the step of nucleic acid extraction from specimens was omitted from this training. Even though approximately 80% of fellows had high competency in nucleic acid extraction and PCR methods prior to the training (Figure 2), 76% who responded to our training evaluation questionnaire preferred both steps (extraction and PCR) to be covered in the training. Two fellows believed that “All steps were necessary, it allows for a refresher of these steps and an opportunity to operate instruments different from the ones in home institutions” and “the best way to learn is through repetition”. However, others would have preferred more time to be allocated to library preparation and sequencing. It seems helpful for fellows to be consulted on the training content and agenda for the programme to have the greatest impact on the fellows in attendance.

The other important constraint faced during the training was performing the wet-laboratory components in groups, such that it was not possible for us to individually complete the protocols. This was one of the main factors that limited our confidence to effectively replicate and teach these methods to others. The ideal scenario would be for each to have a workstation (or groups of up to three fellows) to execute the protocol independently with supervision from trainers. Additionally, it would facilitate learning of the dry-lab methods if the institution provides computers ready for use with compatible specifications and analysis software pre-installed. However, we recognise these limitations are difficult to overcome with constraints on the equipment and space available.

An important gap that we identified is that there was limited room for mentorship and supervision from the trainers. We recommend creating a platform for trainers to follow up with us to monitor our performance and help us to further grow our competencies so that we can in turn share our skills locally with others. We can benefit greatly from continued learning after the training via online forums, periodic webinars, or access to updated learning materials.

Other specific suggestions for improvements are to provide more theoretical background on the principles of the NGS technology and wet-lab protocols to ground fellows who had limited experience in molecular techniques; structure the wet-lab teaching such that a presentation of the overview of the protocol describing the different steps and the corresponding rationale used to adapt protocols is provided ahead of practical work; and improve communication of logistics throughout the training period.

### 3.3. Fellows’ Knowledge Transfer at Home Institutes

#### 3.3.1. Knowledge and Skills Sharing

Upon returning home, we are keen to share our newly gained knowledge and protocols with colleagues at our respective institutions. Further dissemination of these skills will help us to build competent professionals who may be relied upon in outbreak investigations. We plan to share the training material via formally organised internal workshops, which will equally involve the theory and practical components, as was done at the CERI laboratory. We will also share our new knowledge with colleagues in less formal ways, such as during one-on-one conversations in the laboratory. We are confident that any assistance required from CERI and KRISP regarding the in-house training will be duly observed, for example, sharing of updated protocols.

#### 3.3.2. Implementation of Learnt Skills and Knowledge Transfer by Dr. Molalegne Bitew

Dr. Bitew is a Principal Researcher and the Director of the Health Biotechnology Directorate, Bio and Emerging Technology Institute (BETin), in Addis Ababa, Ethiopia. Here, he briefly describes the outcomes of his attendance at this training:

The training was exceptional and timely for BETin of Ethiopia, as presently, the country has just established the third national sequencing facility to serve a population of 120 million. After attending the training, BETin implemented the relevant protocols at the sequencing facility with good results. For example, the facility has adopted the COVID-19 and MTB sequencing protocols and has sequenced 200 SARS-CoV-2 samples, with 139 sequences submitted to GenBank [23]. Furthermore, knowledge and skills were transferred by hosting a genomics and bioinformatics training event at BETin, from 24 to 28 July 2023, for 15 participants from local universities and research institutes in Ethiopia, Sudan, Somalia, and Djibouti. This training was co-organised by Armauer Hansen Research Institute (AHRI) and supported by BETin, the International Centre for Genetic Engineering and Biotechnology (ICGEB), the BiotechNet project, and the Italian Agency for Development Cooperation (AICS). The in-house training will have a ripple effect in upskilling African genomics scientists. Lastly, BETin and CERI have forged a bond for continual collaboration. The two institutions are currently sharing sequencing know-how and collaborating on a research project investigating the multidrug resistance of MTB samples. It is anticipated that this collaboration will continue as new investigations arise.

### 3.4. The Way Forward for Africa: Better Together

This training served to improve pathogen genomic surveillance responses to combat the current disease burden and future emerging disease threats within Africa. We believe the appropriate avenue to consolidate the skills transfer is by integrating the learnt expertise at our home institutions, but also, where possible, by growing new sequencing facilities and bioinformatics units in prominent research institutions in our respective countries. New genomics centres may be disease- or programme-independent and support all pathogen genomics activities at the national level. Ideally, these centres will offer holistic services of efficient pathogen genomics laboratory systems, computational infrastructure, and analytics of the generated data. These centres would service all genomic sequencing needs of the regions in which they are based. With the implementation of simple, interoperable, and affordable tools, genomic surveillance architecture may be developed in a sustainable manner in our countries.

Disease knows no borders, so for the African continent to implement effective disease surveillance, we must strengthen and prioritise partnerships within Africa. The COVID-19 pandemic has taught us a lot in this regard. To develop a partnered continental genomic surveillance system, we envision a specialised multi-sector network of public health and research institutions spanning national and regional levels, such as that of the Africa CDC’s Africa PGI. Within this network, partners work to grow and consolidate technical, financial, and infrastructural resources to collaboratively share specimens, protocols, data, and know-how for rapid disease outbreak investigations. Via these collaborative and pooling strategies, we may begin to overcome common challenges faced by individual institutions in Africa. For example, several institutions grapple with insufficient computational resources and data storage infrastructure, which may be ameliorated by well-resourced partnering institutions with experience in establishing computational systems.

Within this network, we may be able to develop and enforce rigorous quality standards for pathogen genomics laboratories to generate accurate, reliable, and timely data for research and public health decision-making use. Adherence to national and international practices regarding data generation, sharing, and access could be enforced. Based on national policies, genomic data generated within the network would be utilised locally but with the strong encouragement of sharing the data with the global community for timely public health action. Lastly, the network would be well-positioned to initiate further training workshops and conferences for the continuous professional development of genomics and bioinformatics practitioners in Africa. This will facilitate the network to stay abreast of advancements in the field.

African representativity is sorely lacking in human genetic and genomic studies, with the underrepresented populations not receiving the benefits of this research, such as an improved understanding of disease aetiology, drug design, disease diagnosis, and better clinical care [1]. However, the development of a collaborative African pathogen genomics network will allow the scientific community to work together to devise and implement impactful public health actions relevant to African populations. We recognise CERI, KRISP, and CLIMADE as pioneer institutions and consortiums working to grow this network across Africa.

## 4. Conclusions

Genomic methods have proved invaluable for the detection and diagnosis of infectious diseases and are particularly useful in outbreak settings to understand the underlying transmission dynamics for an appropriate public health response. It is imperative to build capacity in these advanced techniques for African scientists to understand and mitigate disease outbreaks. Generally, the training has equipped the fellows with useful skills and techniques that will, in the long term, transform the current methods for rapid characterisation of emerging pathogens. The training was timely, as some institutions are imminently establishing regional sequencing facilities. With this, there is sure to be ongoing collaboration among all participating institutions. An important limitation to note was the need for a longer training period for all fellows to feel adequately trained on both the wet- and dry-lab teachings, but more so for the computationally intensive bioinformatics sessions, highlighting the need for broader and more rigorous investment in the teaching of computational skills at all levels across Africa. Additionally, the training was sufficient as a ‘Train-the-Trainer’ programme for fellows with previous experience in sequencing and bioinformatics methods. However, it needs to be expanded for those with limited prior experience to feel sufficiently equipped to teach the content themselves. Critical evaluation of the training revealed the need for a formalised mechanism for trainers to follow up with fellows after the training for continued learning and support. We recommend that the training programme be continued as a form of professional development to continuously build genomics capacity in Africa. Hence, sponsors should not withhold their support for this programme; it is an innovative upskilling for African genomics and bioinformatics.

## Figures and Tables

**Table 1:** Time allocation per training topic is partitioned into wet-laboratory, computer-based bioinformatics components, science communication for public health, and scientific seminars.

Training Topics	Time Allocated (h)
Wet-Laboratory Training Components	
DNA/RNA amplification	6.5
Library preparation	19.5
Sequencing	8
Adapting protocols to other pathogens	3
Illumina sequencing technology	2.5
Recap and Q&A	3
Bioinformatics Training Components	
Introduction to Unix	1.5
Introduction to bash scripting	1.5
Introduction to R and RStudio	2
Base-calling and assembly	1.5
Sequence assembly and analysis	6
Sequence quality control	2
Sequence submission to public databases	1.5
Basic phylogenetics	5
Recap and Q&A	3
Science Communication for Public Health	1.5
Scientific Seminars	12

## Data Availability

Apart from the data presented in this manuscript, other data supporting the reported results can be found in the Supplementary Materials (S1 and S2).

## Supplementary Materials

The following supporting information can be downloaded at: https://www.mdpi.com/article/10.3390/tropicalmed10040090/s1, S1: S1_Fellows training report_The Google Form; S2: S2_Fellows training report_The Response.
