# Supplementary material for: Building Pathogen Genomic Sequencing Capacity in Africa: Centre for Epidemic Response and Innovation Fellowship
Source: Trop Med Infect Dis. 2025 Mar 31;10(4):90. doi: 10.3390/tropicalmed10040090 (PMC12030795; doi:10.3390/tropicalmed10040090)
Supplement: Supplementary file 1 [file tropicalmed-10-00090-s001.zip › S1_Fellows training report_The Google Form.pdf]

CERI April Fellows training report

Please complete this form with critical thoughts/analysis and reflections of the training. Please be honest and list criticisms and limitations of the training. It is helpful for CERI to receive honest feedback in order to improve future training events. You will remain anonymous when completing this form.

Please complete this form by Monday, 11 December 2023.

* Indicates required question

1. Do you feel this training will transform the way you and your institution will monitor disease outbreaks? Please explain why. *

____
____
____
____
____

2. Was there anything new and different about this training compared to previous trainings you have attended? What did you think were the key features of the training that were different from previous training you have attended? Were there key aspects of the training content, the delivery of the content, the environment etc. that you believe other training initiatives should try to replicate? *

____
____
____
____
____

3. What were the elements of the training that you think could be improved or were not important and could be omitted? *

4. Do you think your institution will collaborate with CERI in the future? If yes, please explain why you are keen to collaborate? *

5. The workshop included attending the opening of Stellenbosch University BMRI building function and the scientific talks relating to that event. Do you think this was useful? Would you have preferred to focus solely on the training? Did you think attending these events contributed positively or negatively to the training? *

6. For the wet lab components of the training, what did you think of the balance of theory and practical hands-on training? *

7. Do you feel that you would have gained much running through the nucleic acids extraction and PCR steps of the protocols in the training? Or could all of these steps have been omitted from the training as you were already familiar with them? *

8. The learning objectives for the training were: *
1. For fellows to develop their skills in wet-lab genomic data production, bioinformatics and phylogenetic data analysis and epidemiology.
2. Develop these skills specifically for sequencing of SARS-CoV-2 genomes and adapting the COVIDSeq protocol, sequencing MTB and cholera genomes. And the bioinformatics that follows the sequencing.

Do you think these learning objectives were met? Please explain why you think they were or were not met.

9. What do feel were the benefits of in-person training (as opposed to virtual/online training)? *

10. Please list and describe any limitations of the training *

11. Please describe how these limitations/problems may be improved *

12. Do you feel the training was sufficient for you to train others on this content? *

This content is neither created nor endorsed by Google.

Google Forms
